# BMP-IHH-mediated interplay between mesenchymal stem cells and osteoclasts supports calvarial bone homeostasis and repair

**DOI:** 10.1038/s41413-018-0031-x

**Published:** 2018-10-17

**Authors:** Yuxing Guo, Yuan Yuan, Ling Wu, Thach-Vu Ho, Junjun Jing, Hideki Sugii, Jingyuan Li, Xia Han, Jifan Feng, Chuanbin Guo, Yang Chai

**Affiliations:** 1 Center for Craniofacial Molecular Biology, University of Southern California, Los Angeles, CA USA; 2 Department of Oral and Maxillofacial Surgery, Peking University School and Hospital of Stomatology, Beijing, China; 3 In Vitro Cell Research, LLC, Englewood, NJ USA; 4 State Key Laboratory of Oral Diseases, West China Hospital of Stomatology, Sichuan University, Chengdu, China; 5 Division of Endodontology, Kyushu University Hospital, Kyushu University, Fukuoka, Japan

## Abstract

Calvarial bones are connected by fibrous sutures. These sutures provide a niche environment that includes mesenchymal stem cells (MSCs), osteoblasts, and osteoclasts, which help maintain calvarial bone homeostasis and repair. Abnormal function of osteogenic cells or diminished MSCs within the cranial suture can lead to skull defects, such as craniosynostosis. Despite the important function of each of these cell types within the cranial suture, we have limited knowledge about the role that crosstalk between them may play in regulating calvarial bone homeostasis and injury repair. Here we show that suture MSCs give rise to osteoprogenitors that show active bone morphogenetic protein (BMP) signalling and depend on BMP-mediated Indian hedgehog (IHH) signalling to balance osteogenesis and osteoclastogenesis activity. IHH signalling and receptor activator of nuclear factor kappa-Β ligand (RANKL) may function synergistically to promote the differentiation and resorption activity of osteoclasts. Loss of *Bmpr1a* in MSCs leads to downregulation of hedgehog (Hh) signalling and diminished cranial sutures. Significantly, activation of Hh signalling partially restores suture morphology in *Bmpr1a* mutant mice, suggesting the functional importance of BMP-mediated Hh signalling in regulating suture tissue homeostasis. Furthermore, there is an increased number of CD200+ cells in *Bmpr1a* mutant mice, which may also contribute to the inhibited osteoclast activity in the sutures of mutant mice. Finally, suture MSCs require BMP-mediated Hh signalling during the repair of calvarial bone defects after injury. Collectively, our studies reveal the molecular and cellular mechanisms governing cell–cell interactions within the cranial suture that regulate calvarial bone homeostasis and repair.

## Skull bone remodeling: Cranial suture cross-talk

Understanding the signaling mechanisms regulating cells in cranial sutures could help develop strategies for repairing skull defects or fractures. Little is known about how osteoblasts, osteoclasts and mesenchymal stem cells (MSCs) in cranial sutures regulate the homeostasis and repair of skull bones. Yang Chai at the University of Southern California, United States, and colleagues show that preventing the expression of bone morphogenetic protein receptor type IA (*Bmpr1a*) in MSCs leads to defective cranial sutures in which osteogenic activity is increased and osteoclast activity is reduced. Stimulating the Hedgehog signaling pathway not only partially rescued the defective sutures but also promoted skull bone healing after injury in *Bmpr1a* mutant mice, highlighting the importance of BMP-mediated Hedgehog signaling for balancing skull bone formation and resorption.

## Introduction

Adult mesenchymal stem cells (MSCs) are undifferentiated multipotent cells that were first identified in the bone marrow but are also present in many other tissues, such as skeletal muscle, placenta, dental pulp, adipose tissue, and cranial sutures.^1–3^ In adult organs, stem and progenitor cells replenish tissues for homeostasis and in response to injury. Gli1 has been proposed to be a marker for MSCs in various organs, including the kidney, lung, liver, heart, tooth, and bone.^4–8^ Recently, it was shown that Gli1+ cells within the cranial suture mesenchyme represent the main MSC population for craniofacial bones and are activated quickly after injury to give rise to craniofacial bones.^3,5,9^

Sutures are fibrous joints in the skull that function as the growth centers of bone formation. During normal postnatal development in humans, cranial sutures remain in a patent, unossified state, while new intramembranous bone is formed at the edges of the osteogenic fronts.^10,11^ The bone remodelling process is maintained by the balance between osteoblast-driven bone formation and osteoclast-driven bone resorption. Osteoclastogenic activity along the osteogenic front is also involved in the regulation of suture patency.^12^ In mice, the posterior frontal suture typically fuses around three weeks after birth, but it exhibits persistent patency in mice lacking osteoprotegerin (OPG), which inhibits osteoclastogenesis by antagonising receptor activator of nuclear factor kappa-B ligand (RANKL).^13^ Moreover, downregulation of another osteoclast regulator, receptor activator of nuclear factor kappa-B (RANK), also results in increased bone formation at the suture.^14^

In the suture, osteoblasts at the osteogenic front and MSCs in the midline are in close proximity during the intramembranous ossification process.^3,15^ Although osteoclasts are present in the suture, their regulatory mechanism has yet to be elucidated. Furthermore, the existence of osteoclasts in the suture provides the opportunity to explore the relationship between suture MSCs, osteoblasts, and osteoclasts. A clear understanding of the relationship among these cells will provide crucial information regarding the dynamic tissue homeostasis of cranial bones and may provide important insights into long bone homeostasis, osteogenic-related diseases such as craniosynostosis, and injury healing.

Previous studies have indicated that BMPR1A is important for tissue homeostasis. In humans, mutation of *BMPR1A* leads to the development of noncancerous growths called hamartomatous polyps in the gastrointestinal tract, known as juvenile polyposis syndrome.^16^ Deletion of *Bmpr1a* in hair follicle stem cells in mice disrupts the hair follicle recycling process.^17,18^ Loss of *Bmpr1a* in differentiated osteoclasts, osteoblasts, or cartilage results in disruption of bone remodelling or growth activities.^19–23^ Expression of the bone morphogenetic protein (BMP) antagonist noggin is correlated with patent sutures;^24^ conversely, increased BMP signalling due to constitutively active *Bmpr1a* in neural crest cells leads to craniosynostosis.^25^ Taken together, these findings suggest that BMPR1A can affect homeostasis in different systems; however, its putative role in regulating the interaction between MSCs and other cells within the suture remains unclear.

In this study, we investigated the role of MSCs and osteoclasts in suture homeostasis and injury repair. Our data showed that Gli1+ MSCs give rise to osteoprogenitors that display active BMP signalling activity within the cranial suture. Conditional inactivation of *Bmpr1a* in Gli1+ MSCs resulted in reduced hedgehog (Hh) signalling and narrowing of the suture due to an imbalance between osteogenic and osteoclastogenic activity. In parallel, in an in vitro osteoclastogenesis assay with bone marrow-derived monocytes/macrophages (BMMs), we found that Indian Hh (IHH) signalling and RANKL may function synergistically to help restore the activity of osteoclasts in *Bmpr1a* mutant mice. Significantly, activation of Hh signalling resulted in a partial rescue of sagittal sutures in *Gli1*-*Cre*^*ERT2*^;*Bmpr1a*^*fl/fl*^ mice. Moreover, loss of *Bmpr1a* also led to enhanced CD200 expression, inhibiting the activation of osteoclasts. In injury models, loss of *Bmpr1a* in Gli1+ MSCs resulted in decreased healing of the calvarial bone injury, but upregulation of Hh signalling promoted the healing of calvarial bone in *Gli1-Cre*^*ERT2*^;*Bmpr1a*^*fl/fl*^ mice. Collectively, our findings reveal the cellular and molecular regulatory mechanisms governing the BMP-IHH signalling-mediated interplay among MSCs, osteoblasts, and osteoclasts that helps to maintain calvarial tissue homeostasis and repair.

## Results

### BMP signalling in Gli1+ suture MSC-derived osteoprogenitors

**Fig. 1:**
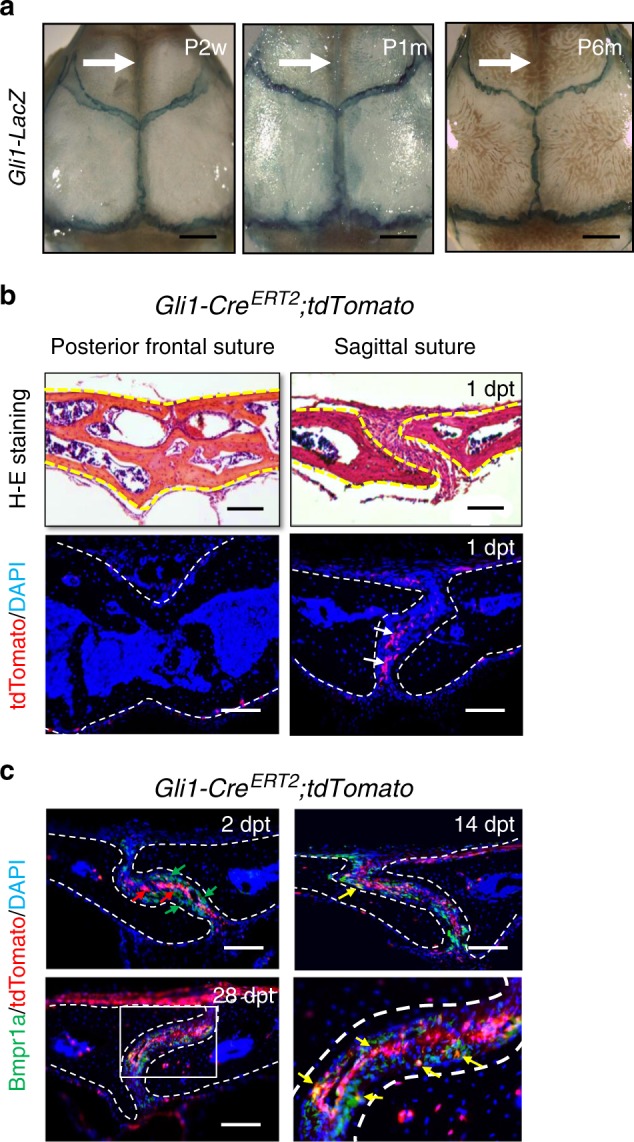
BMP signalling in Gli1+ lineage-derived osteoprogenitors in adult sutures. **a** Whole-mount LacZ staining (blue) of calvarial bones from *Gli1-LacZ* mice at postnatal 2 weeks (P2w), 1 month (P1m), and 6 months (P6m). The arrows indicate the posterior frontal suture. **b** H-E staining and tdTomato (red) visualisation of the posterior frontal suture and sagittal suture of *Gli1-Cre*^*ERT2*^*;tdTomato* mice 1 day post induction with tamoxifen (1dpt). Arrows indicate Gli1+ cells. **c** Bmpr1a immunostaining (green) and tdTomato visualisation (red) of the sagittal suture mesenchyme of *Gli1-Cre*^*ERT2*^*;tdTomato* mice 2 days (2 dpt), 14 days (14 dpt), and 28 days (28 dpt) post induction. Red arrows indicate Gli1+ cells; green arrows indicate Bmpr1a+ cells; yellow arrows indicate co-localization. The boxed area is shown at higher magnification to the right. Broken lines indicate the outline of the suture. Scale bars in **a**, 1 mm; **b** and **c**, 100 µm

Gli1+ stem cells were detectable along all the cranial sutures in adult mice, except the posterior frontal suture, which was already fused at approximately 1 month of age, consistent with previously published results (Fig. 1a, b).^3^ To investigate the role of BMP signalling in Gli1+ cells in maintaining suture patency and calvarial bone homeostasis, we examined the Bmpr1a expression pattern and compared it with the lineage tracing of Gli1+ cells. We found that 2 days after tamoxifen induction of 1-month-old mice, Bmpr1a+ and Sp7+ cells had similar expression patterns in sutures, but mostly, these patterns did not overlap with that of Gli1+ cells located at the suture midline (Supplementary Fig. 1a, b). At later time points, Gli1-derived cells gradually began to express Bmpr1a (Fig. 1c). Our previous work showed that Gli1+ cells give rise to Sp7+ cells 1 month after induction.^3^ Thus, as Gli1+ stem cells leave their niche and begin to differentiate into osteoprogenitors, they express Bmpr1a and Sp7.

### Loss of *Bmpr1a* in Gli1+ cells results in narrowing of cranial sutures

**Fig. 2:**
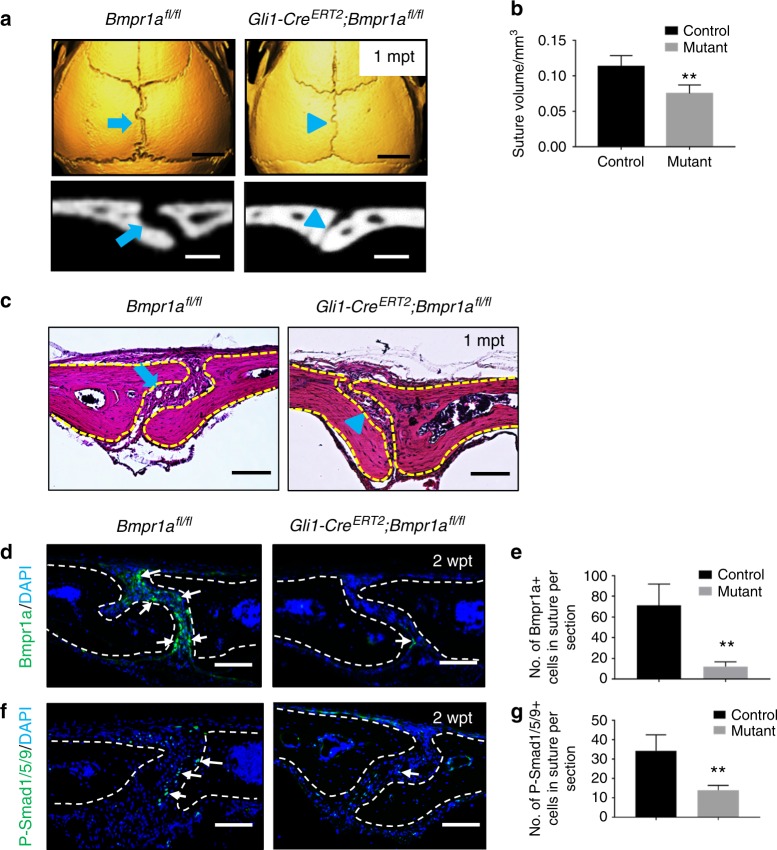
Loss of *Bmpr1a* in Gli1+ lineage-derived cells results in a narrower suture gap. **a** MicroCT scans of sagittal sutures from *Bmpr1a*^*fl/fl*^ (control) and *Gli1-Cre*^*ERT2*^;*Bmpr1a*^*fl/fl*^ (mutant) mice 1 month post tamoxifen induction (1mpt). Coronal sections of the sagittal suture are shown in the bottom panels. **b** Quantitation of the volume of sagittal sutures from six independent *Bmpr1a*^*fl/fl*^ (control) and *Gli1-Cre*^*ERT2*^*;Bmpr1a*^*fl/fl*^ (mutant) mice. **c** H-E staining of sagittal sutures from *Bmpr1a*^*fl/fl*^ and *Gli1-Cre*^*ERT2*^*;Bmpr1a*^*fl/fl*^ mice 1 month post tamoxifen induction (1mpt). The blue arrows in **a** and **c** indicate normal morphology of sagittal sutures from control mice; blue arrowheads in **a** and **c** indicate the narrower suture gap observed in mutant mice. **d** Immunostaining of Bmpr1a (green, indicated by arrows) in the suture mesenchyme of *Bmpr1a*^*fl/fl*^ (control) and *Gli1-Cre*^*ERT2*^*;Bmpr1a*^*fl/fl*^ (mutant) mice 2 weeks post induction (2wpt). **e** Quantitation of Bmpr1a+ cells per section from three independent samples. **f** Immunostaining of P-Smad1/5/9 (green, indicated by arrows) in the suture mesenchyme of *Bmpr1a*^*fl/fl*^ (control) and *Gli1-Cre*^*ERT2*^*;Bmpr1a*^*fl/fl*^ (mutant) mice 2 weeks after induction (2wpt). **g** Quantitation of P-Smad1/5/9+ cells per section from three independent samples. *T* tests were performed. **P < 0.01. Broken lines indicate the outline of the suture. Scale bars in **a** (top panels), 2 mm; **a** (bottom panels), 200 µm; **c**, **d**, and **f**, 100 µm

We hypothesised that BMP signalling is important in suture patency and loss of *Bmpr1a* in the suture would result in a disruption of suture homeostasis. To test this, we generated *Gli1-Cre*^*ERT2*^;*Bmpr1a*^*fl/fl*^ mice in which *Bmpr1a* is lost in cells derived from the Gli1+ population. Indeed, we found that the sagittal suture became much narrower in *Gli1-Cre*^*ERT2*^;*Bmpr1a*^*fl/fl*^ mice compared with control mice 1 month after tamoxifen induction (Fig. 2a). In addition, we found that the volume of the sagittal suture was decreased in *Gli1-Cre*^*ERT2*^;*Bmpr1a*^*fl/fl*^ mice, based on microcomputed tomography (microCT) scan analysis (Fig. 2b, *n* = 6 per group). Histological analysis confirmed that loss of *Bmpr1a* in Gli1+ suture MSCs led to diminished cranial sutures (Fig. 2c). In addition to narrowing of the sagittal suture, our data showed that other cranial sutures, such as the coronal and lambdoid, also became narrower in *Gli1-Cre*^*ERT2*^;*Bmpr1a*^*fl/fl*^ mice (Supplementary Fig. 2a, b). Next, we confirmed that the expression of Bmpr1a and its downstream target, phosphorylated Smad1/5/9, was dramatically decreased in the osteogenic front of sutures in *Gli1-Cre*^*ERT2*^;*Bmpr1a*^*fl/fl*^ mice (Fig. 2d–g).

*Gli1-Cre*^*ERT2*^;*Bmpr1a*^*fl/fl*^ mice exhibited greatly reduced body size and weight 2 months after induction, likely due to their intestinal defects, such as disorganisation of the villi, prominent vacuolation, and accumulation of lipids in the intestines (data not shown). To examine whether the sagittal suture phenotype was secondary to the poor physical condition of *Gli1-Cre*^*ERT2*^;*Bmpr1a*^*fl/fl*^ mice, we conducted an ex vivo kidney capsule assay in which portions of the sagittal sutures from either *Gli1-Cre*^*ERT2*^;*Bmpr1a*^*fl/fl*^ or control mice were implanted in the kidney capsules of healthy recipient mice. The sutures from the mutant mice fused four weeks later, whereas the ones from the controls remained patent (Supplementary Fig. 3). These results suggest that deletion of *Bmpr1*a in Gli1+ cells was responsible for the suture defects.

### Enhanced proliferation and osteogenic differentiation activity after loss of Bmpr1a in Gli1+ cells

**Fig. 3:**
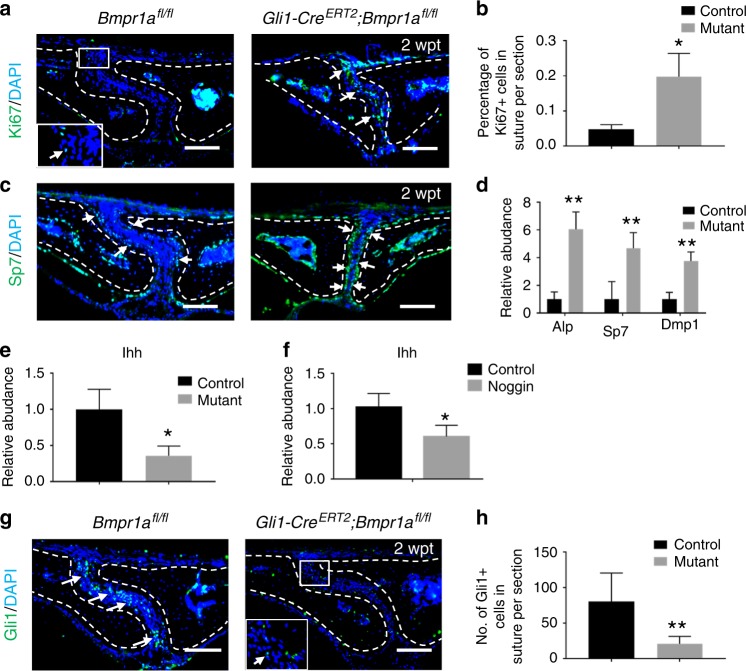
Loss of *Bmpr1a* in cells derived from the Gli1+ lineage results in enhanced proliferation and osteogenic differentiation and weakened IHH signalling. **a** Immunostaining of Ki67 (green, indicated by arrows) in the suture mesenchyme of *Bmpr1α*^*fl/fl*^ (control) and *Gli1-Cre*^*ERT2*^*;Bmpr1α*^*fl/fl*^ (mutant) mice 2 weeks post induction (2wpt). The inset shows the boxed region magnified. **b** Quantitation of percentages of Ki67+ cells in the suture mesenchyme from four independent samples. **c** Immunostaining of Sp7 (green, indicated by arrows) in the suture mesenchyme of *Bmpr1α*^*fl/fl*^ (control) and *Gli1-Cre*^*ERT2*^*;Bmpr1α*^*fl/fl*^ (mutant) mice 2 weeks post induction (2wpt). **d** Real-time PCR of the osteogenic differentiation markers *Alp*, *Sp7*, and *Dmp1* in the suture mesenchyme in five independent samples from *Bmpr1α*^*fl/fl*^ (control) and *Gli1-Cre*^*ERT2*^*;Bmpr1α*^*fl/fl*^ (mutant) mice 2 weeks post induction. **e** Real-time PCR of *Ihh* in the suture mesenchyme in five independent samples from *Bmpr1α*^*fl/fl*^ (control) and *Gli1-Cre*^*ERT2*^*;Bmpr1α*^*fl/fl*^ (mutant) mice 2 weeks post induction. **f** Real-time PCR of *Ihh* in the suture mesenchyme in four independent samples from C57BL/6J mice treated with or without a BMP inhibitor protein (noggin) for 96 h. **g** Immunostaining of Gli1 (green, indicated by arrows) in the suture mesenchyme of *Bmpr1α*^*fl/fl*^ (control) and *Gli1-Cre*^*ERT2*^*;Bmpr1α*^*fl/fl*^ (mutant) mice 2 weeks post induction (2 wpt). The inset shows the boxed region magnified. **h** Quantitation of Gli1+ cells in the suture mesenchyme in three independent samples. *T* tests were performed. *P < 0.05; **P < 0.01. Broken lines indicate the outline of the suture. Scale bars, 100 µm

To investigate the cellular mechanism(s) responsible for the suture defects in *Gli1-Cre*^*ERT2*^;*Bmpr1a*^*fl/fl*^ mice, we first examined proliferation activity in the sutures. We found that proliferation was increased in the sutures of *Gli1-Cre*^*ERT2*^;*Bmpr1a*^*fl/fl*^ mice (Fig. 3a, b). Next, we analysed the effect of loss of *Bmpr1a* in Gli1+ cells on differentiation using Sp7, a marker for osteogenesis. We found that Sp7 expression was increased along the osteogenic fronts of *Gli1-Cre*^*ERT2*^;*Bmpr1a*^*fl/fl*^ mice (Fig. 3c). We also performed quantitative analysis of the RNA expression levels of osteogenic markers, such as *Alp*, *Sp7*, and *Dmp1*, and found that they were all increased in the sutures of *Gli1-Cre*^*ERT2*^;*Bmpr1a*^*fl/fl*^ mice (Fig. 3d), consistent with enhanced osteogenesis.

### Loss of *Bmpr1a* leads to downregulation of Hh signalling

Previously, we found that IHH in the suture helps activate the quiescent suture MSCs.^3^ Hh signalling involves the binding of a Hh ligand to the patched (Ptc) receptor, resulting in activation of *Gli* transcription by the membrane protein smoothened (Smo).^26^ We sought to investigate whether Ihh was secreted from Bmpr1a+ cells in the sutures. Co-localization of Ihh and Bmpr1a in sutures suggested the possibility that Ihh is secreted by Bmpr1a+ osteoprogenitors (Supplementary Fig. 4). We therefore examined *Ihh* gene expression and found that it was downregulated in the sutures of *Gli1-Cre*^*ERT2*^;*Bmpr1a*^*fl/fl*^ mice (Fig. 3e). Local application of noggin, a BMP inhibitor, in the suture also resulted in decreased expression of *Ihh* (Fig. 3f). In addition, Gli1 immunostaining confirmed the decrease of Gli1+ cells in the sutures of *Gli1-Cre*^*ERT2*^;*Bmpr1a*^*fl/fl*^ mice (Fig. 3g, h). Therefore, loss of *Bmpr1a* in Gli1+ cells appears to affect IHH and its downstream pathway.

### Loss of *Bmpr1a* in Gli1+ cells disrupts osteoclastogenic activity, possibly via disruption of RANK, RANKL, and IHH activity

**Fig. 4:**
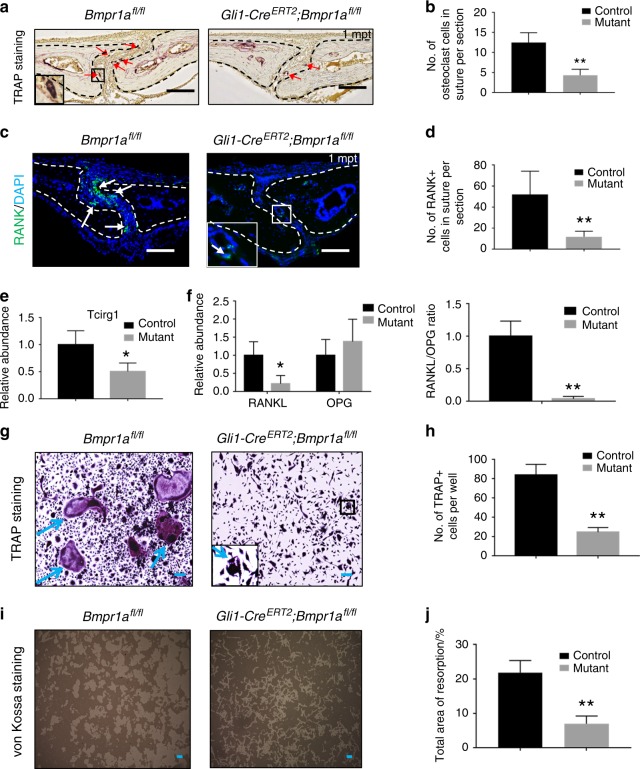
Osteoclastogenic activity is disrupted in *Gli1-Cre*^*ERT2*^*;Bmpr1α*^*fl/fl*^ mice. **a** TRAP staining of the sagittal suture mesenchyme of *Bmpr1α*^*fl/fl*^ (control) and *Gli1-Cre*^*ERT2*^*;Bmpr1a*^*fl/fl*^ (mutant) mice 1 month post induction (1mpt). The inset shows the boxed region magnified. Red arrows indicate mature osteoclasts. **b** Quantitation of TRAP-positive osteoclasts in the suture mesenchyme per section from three independent samples. **c** Immunostaining of RANK (green, indicated by arrows) in the suture mesenchyme of *Bmpr1α*^*fl/fl*^ (control) and *Gli1-Cre*^*ERT2*^*;Bmpr1α*^*fl/fl*^ (mutant) mice 1 month post induction (1mpt). The inset shows the boxed region magnified. **d** Quantitation of RANK+ cells in the suture mesenchyme per section from three independent samples. **e** Real-time PCR of *Tcirg1* in the suture mesenchyme from three independent *Bmpr1a*^*fl/fl*^ (control) and *Gli1-Cre*^*ERT2*^*;Bmpr1a*^*fl/fl*^ (mutant) mice 2 weeks post induction. **f** Real-time PCR of *RANKL* and *OPG* and the ratio of *RANKL/OPG* in the suture mesenchyme from four independent *Bmpr1α*^*fl/fl*^ (control) and *Gli1-Cre*^*ERT2*^*;Bmpr1α*^*fl/fl*^ (mutant) mice 1 month post induction. **g** TRAP staining of osteoclasts induced from BMMs of *Bmpr1α*^*fl/fl*^ (control) and *Gli1-Cre*^*ERT2*^*;Bmpr1α*^*fl/fl*^ (mutant) mice 2 weeks post induction. The cells were cultured with M-CSF for 3 days and then with RANKL for another 5 days. The inset shows the boxed region magnified. Blue arrows indicate mature osteoclasts. **h** Quantitation of multinucleated TRAP-positive osteoclasts per well from four independent experiments. **i** Resorption activity assay (von Kossa staining) of osteoclasts induced from BMMs of *Bmpr1α*^*fl/fl*^ (control) and *Gli1-Cre*^*ERT2*^*;Bmpr1α*^*fl/fl*^ (mutant) mice 2 weeks post induction, after culture with M-CSF for 3 days and then with RANKL for another 5 days. **j** Quantitation of resorption ratio per well from four independent experiments. *T* tests were performed. *P < 0.05; **P < 0.01. Broken lines indicate the outline of the suture. Scale bars, 100 µm

Based on the narrowing of the sutures in *Gli1-Cre*^*ERT2*^;*Bmpr1a*^*fl/fl*^ mice, we hypothesised that osteoclastogenic activity might also be disrupted. Consequently, bone turnover at the osteogenic front of the affected suture would be decreased. To test our hypothesis, we first examined the expression of tartrate-resistant acid phosphatase (TRAP), a marker highly expressed by osteoclasts, and RANK, expressed primarily on monocytes/macrophages/osteoclastic precursors. We found that both TRAP and RANK staining were decreased in the sagittal sutures of *Gli1-Cre*^*ERT2*^;*Bmpr1a*^*fl/fl*^ mice (Fig. 4a–d). To confirm the alteration of osteoclast activity in sutures of *Gli1-Cre*^*ERT2*^;*Bmpr1a*^*fl/fl*^ mice, we also analysed T-cell immune regulator 1 (Tcirg1) expression. Tcirg1 resides in the ruffled border of osteoclasts and controls osteoclast-mediated extracellular acidification.^27^ *Tcirg1* gene expression was also decreased in the sagittal sutures of *Gli1-Cre*^*ERT2*^;*Bmpr1a*^*fl/fl*^ mice (Fig. 4e). The ratio of RANKL to OPG is considered the main regulator of the bone remodelling process, and its imbalance can lead to the loss (osteoporosis) or gain (osteopetrosis) of bone density.^14^ Therefore, we quantitatively analysed the expression of *RANKL* and *OPG* in the sagittal suture and found that *RANKL* expression was significantly decreased in *Gli1-Cre*^*ERT2*^;*Bmpr1a*^*fl/fl*^ mice, although *OPG* expression appeared unaffected (Fig. 4f). Thus, the *RANKL/OPG* ratio was decreased, consistent with a decrease in osteoclast activity (Fig. 4f). We then analysed osteoclast activity in vitro by isolating BMMs from femurs and tibiae of control and *Gli1-Cre*^*ERT2*^;*Bmpr1a*^*fl/fl*^ mice and culturing them with macrophage colony-stimulating factor (M-CSF) and RANKL to promote osteoclastogenesis. BMMs from control mice were able to differentiate into mature osteoclasts, based on TRAP staining, whereas the osteoclast differentiation potential of BMMs was weakened in *Gli1-Cre*^*ERT2*^;*Bmpr1a*^*fl/fl*^ mice (Fig. 4g, h). Similarly, resorption ability, a hallmark of osteoclasts, was also decreased in BMMs from *Gli1-Cre*^*ERT2*^;*Bmpr1a*^*fl/fl*^ mice based on von Kossa staining (Fig. 4i, j).

### Activation of Hh signalling restores osteoclast resorption activity and partially rescues suture morphology in *Gli1-Cre*^*ERT2*^*;Bmpr1α*^*fl/fl*^ mice

**Fig. 5:**
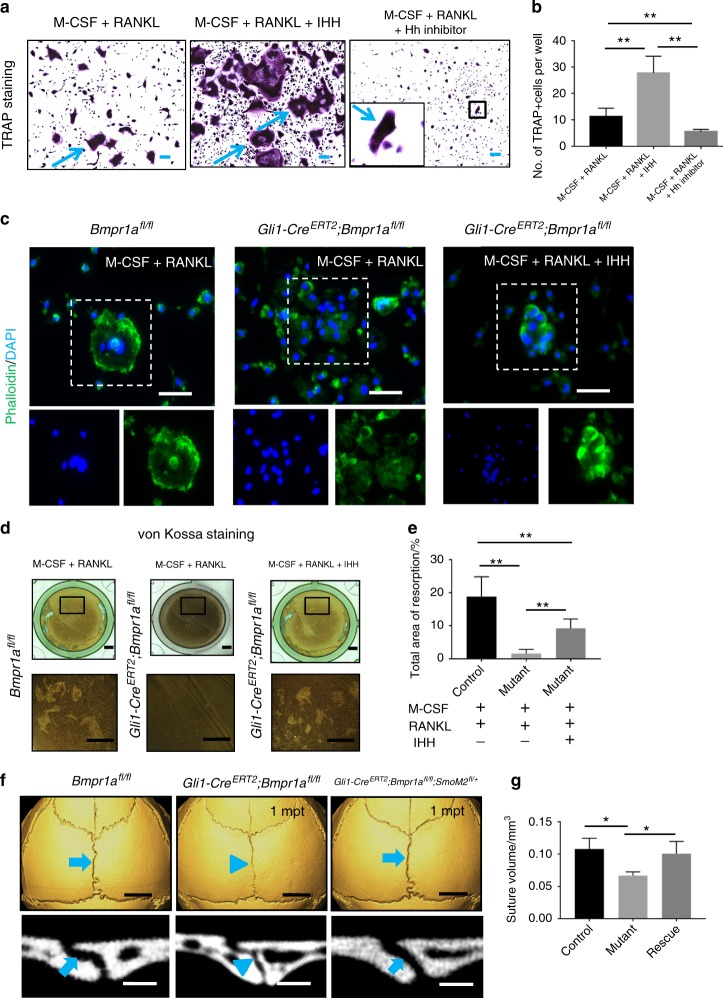
Activation of hedgehog signalling partially restores osteoclast resorption activity and suture gap width in *Gli1-Cre*^*ERT2*^*;Bmpr1α*^*fl/fl*^ mice. **a** TRAP staining of osteoclasts induced from BMMs of 4-week-old C57BL/6J mice, after culture with M-CSF for 3 days and then with RANKL alone or RANKL plus either IHH or hedgehog inhibitor for another 5 days. The inset shows the boxed region magnified. Blue arrows indicate mature osteoclasts. **b** Quantitation of multinucleated TRAP+ osteoclasts per well in six independent experiments. **c** Phalloidin (green) and DAPI (blue) staining of osteoclasts induced from BMMs of *Bmpr1α*^*fl/fl*^ and *Gli1-Cre*^*ERT2*^*;Bmpr1α*^*fl/fl*^ mice 2 weeks post induction, after culture with M-CSF for 3 days and then with RANKL or RANKL plus IHH for another 5 days. Boxes indicate multinucleated cells shown with single staining in the lower panels. **d** Resorption activity assay (von Kossa staining) of osteoclasts induced from BMMs of *Bmpr1α*^*fl/fl*^ (control) and *Gli1-Cre*^*ERT2*^*;Bmpr1α*^*fl/fl*^ (mutant) mice 2 weeks post induction, after culture with M-CSF for 3 days and then with RANKL or RANKL plus IHH for another 5 days. **e** Quantitation of resorption ratio per well in six independent experiments. **f** MicroCT scans of sagittal sutures from *Bmpr1a*^*fl/fl*^ (control), *Gli1-Cre*^*ERT2*^*;Bmpr1a*^*fl/fl*^ (mutant), and *Gli1-Cre*^*ERT2*^*;Bmpr1a*^*fl/fl*^*;SmoM2*^*fl/+*^ (rescue) mice 1 month post induction (1mpt). Coronal sections of the sagittal suture are shown in the bottom panels. Blue arrows indicate normal morphology of sagittal sutures of control and rescue mice; blue arrowheads indicate the narrower suture gap of mutant mice. **g** Quantitation of the volume of sagittal sutures from six independent samples. ANOVA was performed. *P < 0.05; **P < 0.01. Scale bars in **a** and **c**, 100 µm; **d**, 1 mm; **f** (top panels), 2 mm; **f** (bottom panels), 200 µm

Previous studies have reported that sonic Hh (SHH) can help promote the activation of osteoclasts during malignant tumourigenesis.^28,29^ Although SHH is not expressed in sutures of adult mice, IHH is,^3^ and it functions similarly to SHH.^30^ Therefore, we hypothesised that IHH secreted by osteoprogenitors may affect osteoclasts in the suture. To test this, we cultured BMMs in vitro with IHH or Hh inhibitor (GDC0449). When exogenous IHH was added along with M-CSF and RANKL to promote osteoclastogenesis, differentiation of BMMs into mature osteoclasts was increased, whereas addition of the Hh inhibitor had the opposite effect (Fig. 5a, b). In the absence of RANKL, the combination of M-CSF and IHH failed to induce differentiation of BMMs into mature osteoclasts (Supplementary Fig. 5), suggesting that RANKL and IHH may act synergistically in the promotion and/or maintenance of osteoclast activity.

Next, we analysed whether the addition of IHH could help restore the resorption ability of BMMs from *Gli1-Cre*^*ERT2*^;*Bmpr1a*^*fl/fl*^ mice. The formation of F-actin rings is required for osteoclast resorption.^31^ We found that BMMs from control mice formed F-actin rings that included multiple nuclei, whereas BMMs from *Gli1-Cre*^*ERT2*^*;Bmpr1a*^*fl/fl*^ mice failed to form F-actin rings (Fig. 5c). The addition of IHH partially restored F-actin ring formation in *Gli1-Cre*^*ERT2*^*;Bmpr1a*^*fl/fl*^ cells (Fig. 5c). In addition, resorption ability was also partially rescued after addition of IHH to BMMs from *Gli1-Cre*^*ERT2*^*;Bmpr1a*^*fl/fl*^ mice, based on von Kossa staining (Fig. 5d, e). The restoration of osteoclastogenesis in BMMs from *Gli1-Cre*^*ERT2*^;*Bmpr1a*^*fl/fl*^ mice after addition of IHH indicates that the effect of BMP signalling on osteoclasts may be mediated through IHH. Interaction of IHH and BMP signalling has been shown previously in both intramembranous and endochondral ossification.^32,33^ Based on the effect of IHH on osteoclastogenesis, we investigated its relationship with osteoclasts using X-gal and TRAP double staining of 1-month-old *Ihh-LacZ* mice. We failed to detect co-localization, indicating that IHH is not expressed in osteoclasts (Supplementary Fig. 6).

In addition, we investigated whether constitutive activation of Hh signalling using *SmoM2* would reverse the narrowing of the suture gap in *Gli1-Cre*^*ERT2*^;*Bmpr1a*^*fl/fl*^ mice. One month after induction, the width and volume of the sagittal suture in most *Gli1-Cre*^*ERT2*^;*Bmpr1a*^*fl/fl*^;*SmoM2*^*fl/+*^ mice (7/11) were similar to those of control mice (Fig. 5f, g). Taken together, these results suggest that upregulation of Hh signalling helps partially rescue suture homeostasis in *Bmpr1a* mutant mice.

### Increased CD200+ cells may contribute to the decrease in osteoclastogenic activity due to reduced IHH signalling

**Fig. 6:**
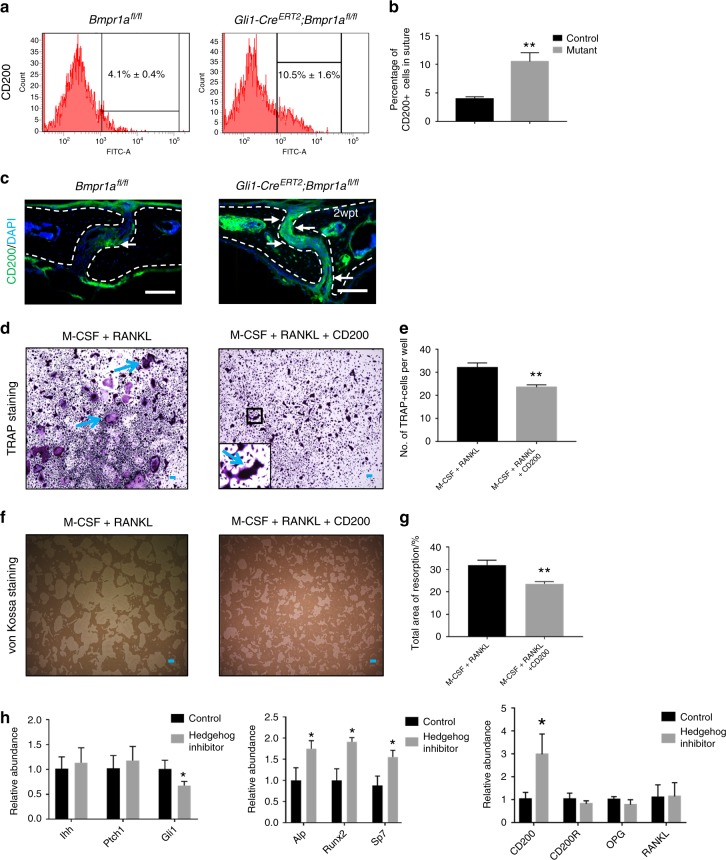
Upregulated CD200 may contribute to decreased osteoclastogenic activity in *Gli1-Cre*^*ERT2*^*;Bmpr1a*^*fl/fl*^ sutures. **a** FACS analysis of CD200 in suture mesenchymal cells collected from *Bmpr1α*^*fl/fl*^ (control) and *Gli1-Cre*^*ERT2*^*;Bmpr1α*^*fl/fl*^ (mutant) mice 2 weeks post induction. **b** Quantitation of the percentages of CD200+ cells in suture mesenchyme from three independent samples. **c** Immunostaining of CD200 (green, indicated by arrows) in the suture mesenchyme of *Bmpr1α*^*fl/fl*^ (control) and *Gli1-Cre*^*ERT2*^*;Bmpr1α*^*fl/fl*^ (mutant) mice 2 weeks post induction (2wpt). Broken lines indicate the outline of the suture. **d** TRAP staining of osteoclasts induced from BMMs of 4-week-old C57BL/6J mice, after culture with M-CSF for 3 days and then with RANKL or RANKL plus exogenous CD200 for another 5 days. The inset shows a magnified view of the boxed region. Blue arrows indicate mature osteoclasts. **e** Quantitation of multinucleated TRAP+ osteoclasts per well in four independent experiments. **f** Resorption activity assay (von Kossa staining) of osteoclasts induced from BMMs of 4-week-old C57BL/6J mice, after culture with M-CSF for 3 days and then with RANKL or RANKL plus exogenous CD200 for another 5 days. **g** Quantitation of the percentage of resorption in four independent experiments. **h** Real-time PCR of hedgehog signalling members (*Ihh*, *Ptch1*, and *Gli1*), osteogenic markers (*Alp*, *Runx2*, and *Sp7*), and osteoclastic-related markers (*CD200*, *CD200R*, *OPG*, and *RANKL*) in the suture mesenchyme of C57BL/6J mice 2 weeks post treatment with or without GDC0449 (hedgehog inhibitor) from five independent samples. *T* tests were performed. *P < 0.05; **P < 0.01. Scale bars, 100 µm

Osteoclastogenesis may also be affected by CD200, an immunoglobulin superfamily member expressed on various types of cells, including MSCs.^31^ Recent studies have reported that CD200 may be a marker for MSC-derived clones with relatively high osteogenic potential.^31,34^ In contrast, the CD200 receptor (CD200R) is expressed on myeloid cells, such as monocytes and macrophages.^35^ We found that the percentage of CD200+ cells in the sagittal suture was higher in *Gli1-Cre*^*ERT2*^;*Bmpr1a*^*fl/fl*^ mice than in control mice (control, 4.1% ± 0.4%; *Bmpr1a* mutant, 10.5% ± 1.6%) (Fig. 6a, b). Increased CD200 expression in the sutures of *Gli1-Cre*^*ERT2*^;*Bmpr1a*^*fl/fl*^ mice was also detectable using immunostaining (Fig. 6c). Next, we cultured BMMs and induced osteoclastogenesis with or without recombinant CD200. We found that exogenous CD200 could block osteoclast differentiation, based on osteoclast cell number and area of resorption activity (Fig. 6d–g).

Because our analysis of *Gli1-Cre*^*ERT2*^;*Bmpr1a*^*fl/fl*^ mice indicated that CD200 and IHH exert opposite effects on osteoclastogenesis, we hypothesised that CD200 may be affected by IHH signalling in the suture niche. We tested this using local application of a Hh inhibitor (GDC0449) on the sagittal suture. After 2 weeks, *Gli1* expression was downregulated, and the expression levels of osteogenic markers, such as *Alp*, *Runx2*, and *Sp7*, were increased (Fig. 6h). Moreover, *CD200* expression was increased nearly threefold, although other osteoclast-related genes (*OPG* and *RANKL*) were unaffected (Fig. 6h). Therefore, the increase of CD200+ cells in suture after loss of *Bmpr1a* in Gli1+ cells may have an effect on osteoclastogenic and osteogenic activity in addition to that resulting from the altered RANKL/OPG ratio and IHH expression.

### Activated Hh signalling promotes the healing of calvarial injuries in *Gli1-Cre*^*ERT2*^;*Bmpr1a*^*fl/fl*^ mice

**Fig. 7:**
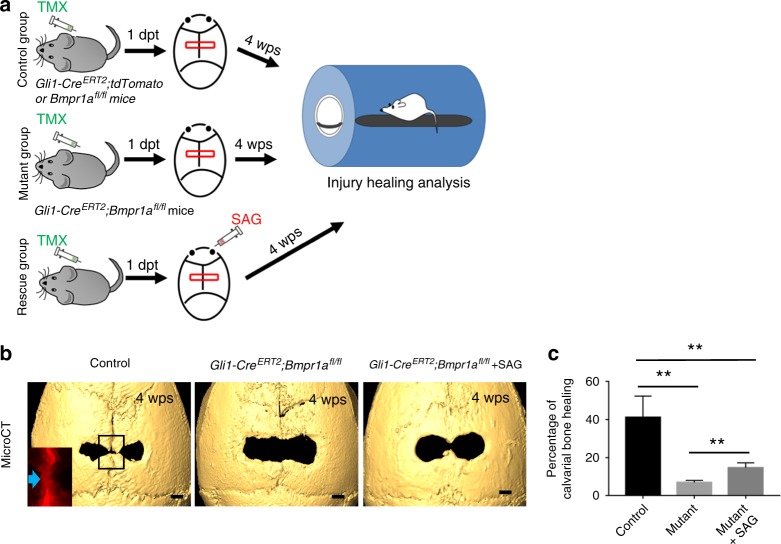
Activated hedgehog signalling promotes the healing of calvarial injuries in *Gli1-Cre*^*ERT2*^*;Bmpr1a*^*fl/fl*^ mice. **a** Schematic diagram depicts the experimental design. Calvarial injuries were created in *Gli1-Cre*^*ERT2*^*;tdTomato* or *Bmpr1a*^*fl/fl*^ (control) and *Gli1-Cre*^*ERT2*^*;Bmpr1a*^*fl/fl*^ (mutant) mice 1 day post induction (1dpt) with tamoxifen (TMX). The red boxes indicate the injury. A hedgehog agonist (SAG) was then injected twice a week into one group of mutant mice, starting at the time of injury (rescue). Skulls were analysed 4 weeks post surgery (4wps) to assess healing. **b** MicroCT analysis of the skull defects in *Gli1-Cre*^*ERT2*^*;tdTomato* (control), *Gli1-Cre*^*ERT2*^*;Bmpr1a*^*fl/fl*^ (mutant), and *Gli-Cre*^*ERT2*^*;Bmpr1a*^*fl/fl*^ mice treated with SAG (rescue) at 4wps. The inset shows tdTomato visualisation of the boxed region from a *Gli1-Cre*^*ERT2*^*;tdTomato* mouse skull. **c** Quantitation of the percentage of the skull injury defect healed after 4 weeks in five independent samples. ANOVA was performed. **P < 0.01. Scale bars, 1 mm

The maintenance of suture homeostasis by MSCs, osteoblasts, and osteoclasts prompted us to investigate their effect during the injury-healing process in the calvaria. Our previous studies showed that sutures hold strong regenerative capacity following calvarial bone injury.^9^ After creating a rectangular defect crossing the sagittal suture, we monitored calvarial bone healing based on visual inspection over time (Fig. 7a). We found that Gli1+ MSCs contributed to the newly formed bone along the sagittal suture. Calvarial bone healing was severely impaired in *Gli1-Cre*^*ERT2*^;*Bmpr1a*^*fl/fl*^ mice (Fig. 7b, c). However, upregulation of Hh signalling using a Hh agonist (SAG) helped partially restore the calvarial bone-healing process after the creation of a calvarial bone defect in *Gli1-Cre*^*ERT2*^;*Bmpr1a*^*fl/fl*^ mice (Fig. 7b, c). Thus, we conclude that Hh signalling may also function in the BMP-mediated calvarial bone defect-healing process.

## Discussion

**Fig. 8:**
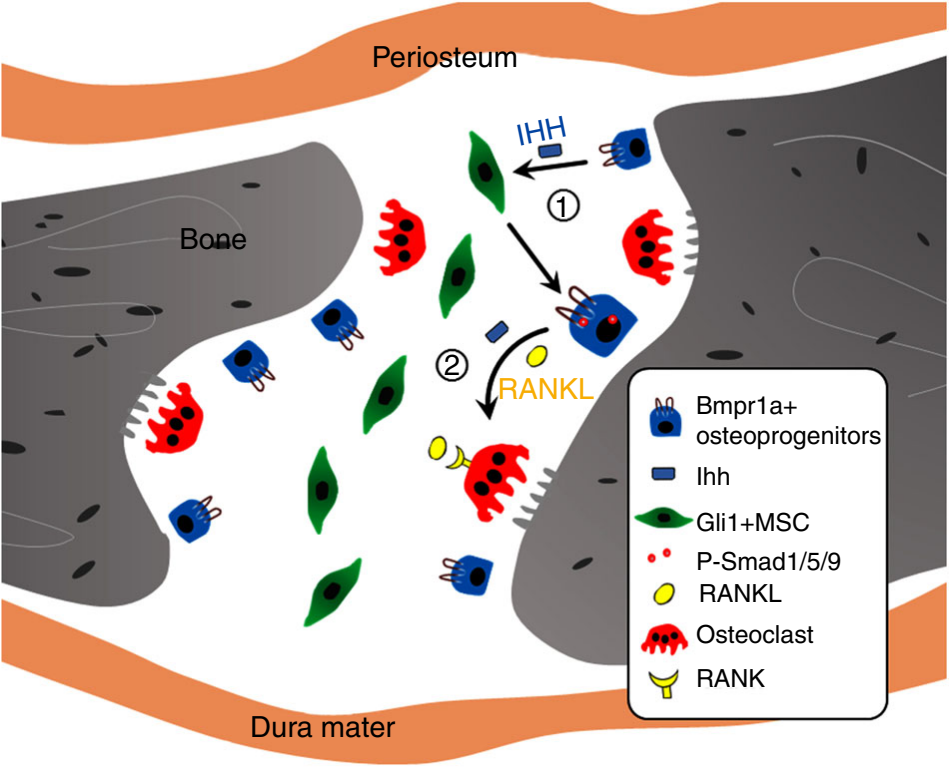
The interplay between mesenchymal stem cells and osteoclasts supports calvarial bone tissue homeostasis. 1 Suture MSCs are activated by IHH secreted by osteoprogenitors, which is dependent on BMP signalling. 2 BMP-mediated IHH signalling and RANKL may function synergistically to promote the differentiation and resorption activity of osteoclasts

Cell–cell interactions play a crucial role in regulating tissue homeostasis. Although the interaction between osteoblasts and osteoclasts is critical in bone tissue remodelling, we have limited information on how MSCs participate in this osteoblast–osteoclast interaction. The proximity of MSCs, osteoblasts, and osteoclasts within the cranial suture renders it a good model for studying the interactions between these three different cell types. In our study, we discovered that loss of *Bmpr1a* from Gli1+ MSC-derived osteoprogenitors results in a narrower suture gap in adult mice due to imbalanced osteogenesis and osteoclastogenesis. Furthermore, decreased osteoclastogenesis in *Bmpr1a* mutant mice is due to downregulation of IHH signalling and osteoclastogenesis regulators (RANKL/OPG ratio), as well as increased CD200 expression. In parallel, our in vitro osteoclastogenesis assays using BMMs revealed that IHH signalling and RANKL function synergistically to promote the differentiation and resorption activity of osteoclasts and that CD200 acts to inhibit osteoclastogenesis. Taken together, our results indicate that suture homeostasis depends on BMP-IHH signalling in MSCs to maintain the balance of MSC-derived osteoprogenitors and their interaction with other neighbouring cells, such as osteoclasts (Fig. 8). This discovery highlights a crucial function of MSCs in mediating the interplay of bone-forming and -resorbing cells in bone tissue homeostasis in adults.

Osteoblasts play dual roles in bone formation, because they help create bone tissue as well as promote the maturation of osteoclasts by secreting RANKL and OPG.^36,37^ Our results demonstrate that osteoclast and osteoblast activity at the osteogenic fronts of a suture helps maintain suture patency via dynamic bone turnover. Previously, in vitro co-culture models have been established to explore the effect of MSCs on osteoclastogenesis. Specifically, co-culture with osteoclast progenitors induces human bone marrow-derived MSCs to secrete cytokines, such as IL6, IL11 leukemia inhibitory factor, and M-CSF, which can promote osteoclast formation.^38^ In contrast, CD200+ cells can block osteoclastogenesis via cell–cell contact.^35^ Furthermore, in vitro studies have shown that CD200+ MSCs can promote osteogenesis.^31,35,39^ We report here that CD200 is upregulated due to the disruption of BMP-mediated IHH signalling. It is plausible that CD200 expression has to be tightly regulated under normal conditions in order to maintain the balance between osteoclastogenesis and osteogenesis in cranial sutures.

Both BMP and IHH have been reported to promote osteogenesis during development and in bone defects or fractures.^40,41^ There is also evidence that BMP-mediated IHH signalling positively regulates osteoprogenitor recruitment to the osteogenic front.^32,42^ In our study, osteogenesis was enhanced even though BMP-mediated IHH signalling was impaired. This is likely due to increased osteoprogenitor cell differentiation and reduced osteoclast activity. It highlights the importance of the interaction between MSCs, osteoprogenitors, and osteoclasts in maintaining suture homeostasis. In parallel, BMP-IHH signalling in osteoprogenitors also appears to be crucial for osteoclast activity in the long bone, as shown in our in vitro cell culture experiments using BMMs. A recent study has shown that Gli1+ cells give rise to bone marrow stromal cells and adipocytes. These Gli1+ cells also express mesenchymal stem/progenitor markers and participate in long bone homeostasis and injury repair.^8^ Our data suggest that a BMP-HH signalling cascade is likely required in osteoprogenitors to regulate osteoclast activity in long bones. Additional studies are required to test whether this interaction between MSCs, osteoprogenitors, and osteoclasts serves as a conserved mechanism in regulating bone tissue homeostasis.

Gli1+ cells in the suture function as typical MSCs in vivo and contribute to the healing of calvarial defects after injury.^3,9,43^ During the injury-healing process, the interaction between MSCs, osteoblasts, and osteoclasts is critical for successful bone repair.^44^ Consistent with previous reports,^45–47^ we found that mice with reduced osteoclast activity show impaired healing ability. Osteoclasts may facilitate suture MSC migration to the defect site by widening the suture gap (our unpublished data). Impaired osteoclast activity that blocks this MSC migration may underlie the reduced capacity for bone regeneration seen here after loss of *Bmpr1a* in Gli1+ cells. Importantly, our study also reveals the molecular mechanism by which the BMP-IHH signalling cascade in MSCs plays a crucial role in regulating osteoclast activity in calvarial bone regeneration.

In summary, our data indicate that MSCs not only serve as a resource for maintaining calvarial tissue homeostasis but also interact with osteoclasts and osteoblasts to achieve their function. Osteoclasts play an important role in maintaining suture patency. BMP-dependent Hh signalling regulates the interplay between suture MSCs and osteoclasts that results in precise regulation of calvarial bone homeostasis and is crucial for injury repair. Future work elucidating the interplay between these cell types may aid in the treatment of craniosynostosis and in stem cell-mediated tissue regeneration to repair skull defects.

## Materials and methods

### Generation of transgenic mice

*Gli1-Cre*^*ERT2*^ (JAX#007913^48^), *tdTomato* (JAX#007905^49^), *Gli1-LacZ* (JAX#008211^50^), *SmoM2*^*fl/fl*^ (JAX #005130^51^), and C57BL/6J (JAX# 000664) mouse lines were obtained from Jackson Laboratory. *Bmpr1a*^*fl/fl*^ and *Ihh-LacZ* mice were kindly provided by Sarah E. Millar (University of Pennsylvania) and Andrew McMahon (University of Southern California), respectively.^52,53^

All mice were housed in pathogen-free conditions and analysed in a mixed background. Ear tags were used to identify mice. Tissue from ear biopsies was lysed by incubation in Direct PCR tail solution (Viagen 102-T) at 55 °C overnight followed by 30 min of heat inactivation at 85 °C. PCR-based genotyping was used to identify the mouse lines (GoTaq Green Master Mix, Promega, and C1000 Touch Cycler, Bio-Rad). Mice were euthanized via carbon dioxide overdose followed by cervical dislocation. All studies were performed with the approval of the Institutional Animal Care and Use Committee of the University of Southern California. All mice were used for analysis without consideration of sex. For induction of Cre lines, tamoxifen (Sigma T5648) was suspended in corn oil (Sigma C8267) at 20 mg·mL^‒1^ and injected intraperitoneally at a dose of 1.5 mg per 10 g body weight for 3 consecutive days.

For the experiments shown in Fig. 1a, heterozygous *Gli1-LacZ* mice were used. Mice were collected at the indicated ages and genotyped.

For the experiments shown in Fig. 1b, c and Supplementary Fig. 1, *Gli1-Cre*^*ERT2*^; *tdTomato* mice were induced at 1 month of age with tamoxifen. Samples were collected at the indicated time points after induction.

For the experiments shown in Figs. 2–6, *Gli1-Cre*^*ERT2*^;*Bmpr1a*^*fl/fl*^ and *Gli1-Cre*^*ERT2*^;*Bmpr1a*^*fl/fl*^;*SmoM2*^*fl/+*^ mice were induced at 1 month of age with tamoxifen and collected at the indicated time points after induction. *Bmpr1a*^*fl/fl*^ or C57BL/6J mice were used as controls. Cells from the sutures were used after digestion by collagenase for flow cytometry analysis. Bone marrow cells from tibia and femur bone marrow were used for analysis of osteoclastogenesis.

For the experiments shown in Fig. 7, *Bmpr1a*^*fl/fl*^ or *Gli1-Cre*^*ERT2*^; *tdTomato* and *Gli1-Cre*^*ERT2*^;*Bmpr1a*^*fl/fl*^ mice were used to generate calvarial defects.

For the experiments shown in Supplementary Figs. 2–4, *Gli1-Cre*^*ERT2*^;*Bmpr1a*^*fl/fl*^ and *Bmpr1a*^*fl/fl*^ mice were euthanized at the indicated time points.

For the experiments shown in Supplementary Fig. 5, cells were obtained from the tibia and femur bone marrow of C57BL/6J mice for analysis of osteoclastogenesis.

For the experiments shown in Supplementary Fig. 6, *Ihh-lacZ* mice were euthanized at the indicated time points.

For the kidney capsule transplantation experiments shown in Supplementary Fig. 3a, sutures collected from 1-month-old *Bmpr1a*^*fl/fl*^ and *Gli1-Cre*^*ERT2*^;*Bmpr1a*^*fl/fl*^ mice after tamoxifen induction for 3 consecutive days were implanted into C57BL/6J mice. The samples were collected 4 weeks later for morphological analysis.

For the experiments shown in Fig. 6h, 200 mmol·L^‒1^ Hh inhibitor (Selleckchem, Vismodegib [GDC0449], S1082) in dimethylsulphoxide was injected locally above the sagittal suture at a dosage of 2.67 μL per 10 g body weight twice a week.

For the experiments shown in Fig. 7b, IHH agonist (SAG dihydrochloride solution, Sigma, SML1314) was dissolved in distilled water at 1 mM and injected intraperitoneally at a dosage of 50 μL per 10 g body weight twice a week.

For the experiments shown in Fig. 3f, BMP inhibitor (noggin, Sigma, SRP4675) was dissolved in phosphate-buffered saline (PBS) at 50 ng/μl and injected locally above the sagittal suture at a dose of 100 μL per mouse.

### MicroCT analysis

Calvaria were dissected and fixed in 4% paraformaldehyde overnight. Samples were radiographed using a SCANCO µCT50 (Scanco V1.28) device at the University of Southern California Molecular Imaging Center. Images were collected at a resolution of 10–30 µm using a 70 kVp and 114 µA X-ray source. AVIZO 9.4.0 (Thermo Fisher Scientific) was used to perform three-dimensional reconstruction. Measurements of sagittal suture volume were performed on segmented parietal bones.

### Histology

Samples were dissected under a stereomicroscope (Leica L2) and fixed in 4% paraformaldehyde at room temperature overnight. Following decalcification in 20% EDTA for 1–2 weeks depending on the mouse age, samples were passed through serial concentrations of ethanol for paraffin embedding. After sectioning at 12–16 μm using a microtome (Leica), haematoxylin and eosin staining was performed on deparaffinized sections following standard procedures.

For cryosections, decalcified samples were dehydrated gradually in 15% sucrose solution for 2–3 h, followed by 30% sucrose for 2–3 h, and 60% sucrose/OCT (Tissue-Tek, Sakura) (1:1) at 4 °C overnight. After being embedded in OCT compound under a stereomicroscope, the samples were frozen in dry ice and sectioned at 12–14 μm thickness using a cryostat (Leica CM1850).

### X-gal staining of whole-mount samples

X-gal staining was performed as described previously.^54^ Briefly, after fixation in 4% paraformaldehyde at room temperature for 30 min followed by three 5-min rinses with PBS at room temperature, samples were incubated overnight in 1 mg/ml X-gal staining solution at 37 °C until colour developed. Next, tissues were fixed in 10% formalin for 1 h at room temperature, washed with 70% ethanol until bleached, and then incubated in fresh 70% ethanol. We monitored *LacZ* expression by looking for the appearance of blue spots on the tissue.

### TRAP staining

The TRAP staining protocol was adapted from a previous report.^55^ EDTA-decalcified tissue cryosections were air-dried at room temperature for 30 min. The slides were rehydrated by three 5-min rinses with PBS and incubated with the TRAP staining solution for 30 min or until the positive colour developed in a 37 °C water bath, according to the manufacturer’s protocol (Sigma-Aldrich, 387A). The slides were counterstained with Fast Green solution for 20 min and mounted with Fluoro-gel medium (Electron Microscopy Sciences, 50–247–04). Multinucleated TRAP-positive osteoclasts (three or more nuclei) were counted using five slides per mouse under a light microscope. Double-labelling analysis of *LacZ* and TRAP was performed by TRAP staining of the X-gal-stained sections.

### Immunostaining

Staining was performed according to standard procedures. Briefly, sections were air-dried for 30 min before removal of OCT via three rinses with PBS. Next, sections were treated with 0.5%–1% Triton100/PBS (Triton100, Sigma, T9284) solution depending on the position of the target gene. After three washes in 0.1% Tween20/PBS (PBST) (Tween20, Sigma, P7949), sections were incubated with a commercial blocking reagent (Abcam, ab126587) for 1 h followed by a primary antibody overnight at 4 °C. Sections were washed three times in PBST and then incubated with the Alexa-conjugated secondary antibody (Invitrogen, A-11008). Antibodies targeting the following proteins were used for immunostaining: Bmpr1a (1:100, Invitrogen, 38–6000), Gli1 (1:500, Novus, NBP1–78259), Ihh (1:50, Abcam, Ab52919), Ki67 (1:100, Abcam, Ab15580), P-Smad1/5/9 (1:500, Cell Signaling, #13820), RANK (1:500, Novus, NBP1–85771), and Sp7 (1:200, Abcam, Ab22552). Alexa Fluor 568 and Alexa Fluor 488 (1:200, Invitrogen) were used for signal detection. For .P-Smad1/5/9, Gli1, and RANK, Alexa Fluor™ 488 Tyramide SuperBoost™ kits (Invitrogen, B40922) were used. 4′,6-diamidino-2-phenylindole (DAPI) (Invitrogen, 62248) was used for counterstaining. Visualisation was performed using a fluorescence microscope (Leica DMI 3000B) with filter settings for DAPI/fluorescein isothiocyanate (FITC)/TRITC.

### Isolation of BMMs

The BMM isolation protocol was adapted from published studies.^45,56^ After dissection of femurs and tibiae, bone marrow cells were flushed out with PBS and transferred into a 50 ml conical tube through a 70-μm cell strainer (Falcon™, 352350). After the bone marrow sample was concentrated, it was resuspended in 1% bovine serum albumin (BSA)/PBS and purified using a density gradient cell separation medium (Ficoll-Paque PREMIUM, 17–5442–02, GE Healthcare Bio-Sciences). The bone marrow cells were washed twice in PBS and resuspended in α-minimum essential medium (α-MEM)/foetal bovine serum (FBS) culture medium (α-MEM [Invitrogen 16000044] supplemented with 2 mmol·L^‒1^ L-glutamine, 100 U·mL^‒1^ penicillin, 100 U·mL^‒1^ streptomycin, and 10% heat-inactivated FBS).

### Osteoclast formation

Briefly, 1 × 10^6^ bone marrow cells were plated in six-well dishes and cultured for 3 days in α-MEM/FBS supplemented with 50 ng·mL^‒1^ M-CSF to produce macrophages, followed by 5 days of culture in α-MEM/FBS with 50 ng·mL^‒1^ M-CSF and 50 ng·mL^‒1^ RANKL to generate mature osteoclasts, following a previously reported protocol.^45^ To assess the effect of CD200, IHH, and Hh inhibitor on osteoclastogenesis, 1 µg·mL^‒1^ CD200 Fc chimaera protein (R&D system, 2724-CD-050), 500 ng·mL^‒1^ IHH (R&D system, 1705-HH-025), or 500 ng/ml Hh inhibitor (Selleckchem, Vismodegib (GDC0449), S1082) was added during the final 5 days of cell culture (Supplementary Fig. 5a). After fixation, cells were stained for TRAP using an acid-phosphatase leucocyte diagnostic kit (Sigma-Aldrich, 387 A) and counterstained with haematoxylin and DAPI. Using a light microscope, multinucleated TRAP-positive osteoclasts (three or more nuclei) were quantitated. The number of osteoclasts was determined using ImageJ software (NIH, Bethesda, MD).

To detect actin ring formation, cells were fixed in 4% paraformaldehyde and permeabilized with 0.5%–1% Triton100/PBS solution for 10 min. After three washes with PBST, slides were incubated with a blocking reagent (Abcam, ab126587) for 1 h and incubated with phalloidin-Alexa 488 (Invitrogen, A12379) for 30 min at room temperature. After three washes with PBST, nuclei were counterstained with DAPI. Cells were imaged using a fluorescence microscope (Leica DMI 3000B) with an attached digital camera.

To determine the resorption activity of osteoclasts, 1 × 10^6^ bone marrow cells were plated in 24-well polystyrene culture dishes precoated with bone substrate (Sigma, Corning^®^ osteo assay, CLS3987) and cultured as described above. After being incubated in 10% bleach solution and washed with distilled water, samples were allowed to air dry for 3–5 h and then counterstained using a von Kossa staining kit (Abcam, 150687) to allow for easier visualisation of the unresorbed substrate. Bright-field microscopy (Keyence microscope, bzx710) was used to visualise the resorbed surface. The resorbed surface area was quantified using ImageJ software.

### Flow cytometry analysis of suture and bone marrow cells

The suture cell collection procedure was adapted from a previous study.^3^ After removal of the periosteum and dura, sagittal sutures were dissected including ∼0.5 mm of the parietal bone immediately adjacent on both sides. Samples were minced and digested using 4 mg/ml Dispase (GIBCO, 17105041) and 4 mg/ml collagenase type I (GIBCO, 17100017) solution at 37 °C for 1 h. Cell suspensions were transferred into a 50 ml conical tube through a 70-μm cell strainer (Falcon™, 352350). After the sample was concentrated, it was treated with lysis buffer (ThermoFisher, HYL250) for 10 min and resuspended in 1% BSA/PBS. Cell suspensions were stained with FITC-conjugated CD200 antibody (1:30, ThermoFisher, MA5–17980) for 30 min at room temperature followed by thorough washing with PBS. Samples were analysed with a BD SORP LSRII Flow Cytometer (BD Bioscience) following standard procedures, as described previously.^3^

### Suture and calvarial bone injury assays

A sagittal incision was created in the midline region of the skull. The scalp was exposed, followed by removal of the periosteum to reveal the sagittal suture. In the calvarial defect model, a rectangular area of bone that transversed the sagittal suture was removed using a round dental bur with a 1-mm diameter. A hand-held drill (NSK Z500) was used. Extreme care was taken to protect the underlying dura. The scalp was then closed with interrupted sutures using 5-0 nylon. The newly formed bone was scanned using microCT and measured by ImageJ software. Healing was analysed according to the following ratio: (initial size of the injury site − final size of the injury site)/(initial size of the injury site).

### Gene expression analysis

An RNeasy Plus Mini Kit (Qiagen, 74134) was used to extract total RNA, and 1 µg of RNA was reverse-transcribed using an iScript cDNA Synthesis Kit (Bio-Rad, 1708891). Real-time quantitative PCR was performed using Soso Fast™ Eva Green^®^ Supermix (Bio-Rad, 1725201) and a CFX 96 thermocycler (Bio-Rad iCycle). GAPDH expression was used to normalise the relative gene expression. Primer sequences are listed in Table 1.

**Table 1:** Primer sequences used for mRNA transcript analysis

Gene name	Forward sequence	Reverse sequence	NCBI gene ID	Primerbank ID
*Alp*	CCAACTCTTTTGTGCCAGAGA	GGCTACATTGGTGTTGAGCTTTT	11647	160333225c1
*Sp7*	ATGGCGTCCTCTCTGCTTG	TGAAAGGTCAGCGTATGGCTT	170574	18485518a1
*Dmp1*	CATTCTCCTTGTGTTCCTTTGGG	TGTGGTCACTATTTGCCTGTG	13406	33469121a1
*Ihh*	CTCTTGCCTACAAGCAGTTCA	CCGTGTTCTCCTCGTCCTT	16147	14149643a1
*Tcirg1*	CACAGGGTCTGCTTACAACTG	CGTCTACCACGAAGCGTCTC	27060	31980624a1
*RANKL*	AGCCGAGACTACGGCAAGTA	AAAGTACAGGAACAGAGCGATG	21943	114842414c1
*OPG*	CCTTGCCCTGACCACTCTTAT	CACACACTCGGTTGTGGGT	18383	113930715c1
*Ptch1*	GCCTTCGCTGTGGGATTAAAG	CTTCTCCTATCTTCTGACGGGT	19206	118130558c1
*Gli1*	CCAAGCCAACTTTATGTCAGGG	AGCCCGCTTCTTTGTTAATTTGA	14632	6754002a1
*Runx2*	GACTGTGGTTACCGTCATGGC	ACTTGGTTTTTCATAACAGCGGA	12393	225690525c1
*CD200*	CTCTCCACCTACAGCCTGATT	AGAACATCGTAAGGATGCAGTTG	17470	31543263a1
*CD200R*	AGGCATTTCCAGTATCACAAGG	CCAATGGCCGACAAAGTAAGG	57781	26354488a1
*GAPDH*	AGGTCGGTGTGAACGGATTTG	GGGGTCGTTGATGGCAACA	14433	126012538c1

### Sample size and statistics

GraphPad Prism 7 software was used for statistical analysis. Two-sided Student’s *T*-tests or analysis of variance were used for statistical analysis to determine significance. A *P*-value < 0.05 was considered significant. *N* = 3 for all experiments except where stated otherwise. For quantifications of all immunostaining experiments, at least five sections per mouse were examined for comparison. Statistical data are presented as the mean ± SD.

## Electronic supplementary material

Supplementary figure 1

Supplementary figure 2

Supplementary figure 3

Supplementary figure 4

Supplementary figure 5

Supplementary figure 6
