# Supplementary figures and images for: BMP-IHH-mediated interplay between mesenchymal stem cells and osteoclasts supports calvarial bone homeostasis and repair
Source: Bone Res. 2018 Oct 17;6:30. doi: 10.1038/s41413-018-0031-x (PMC6193039; doi:10.1038/s41413-018-0031-x)

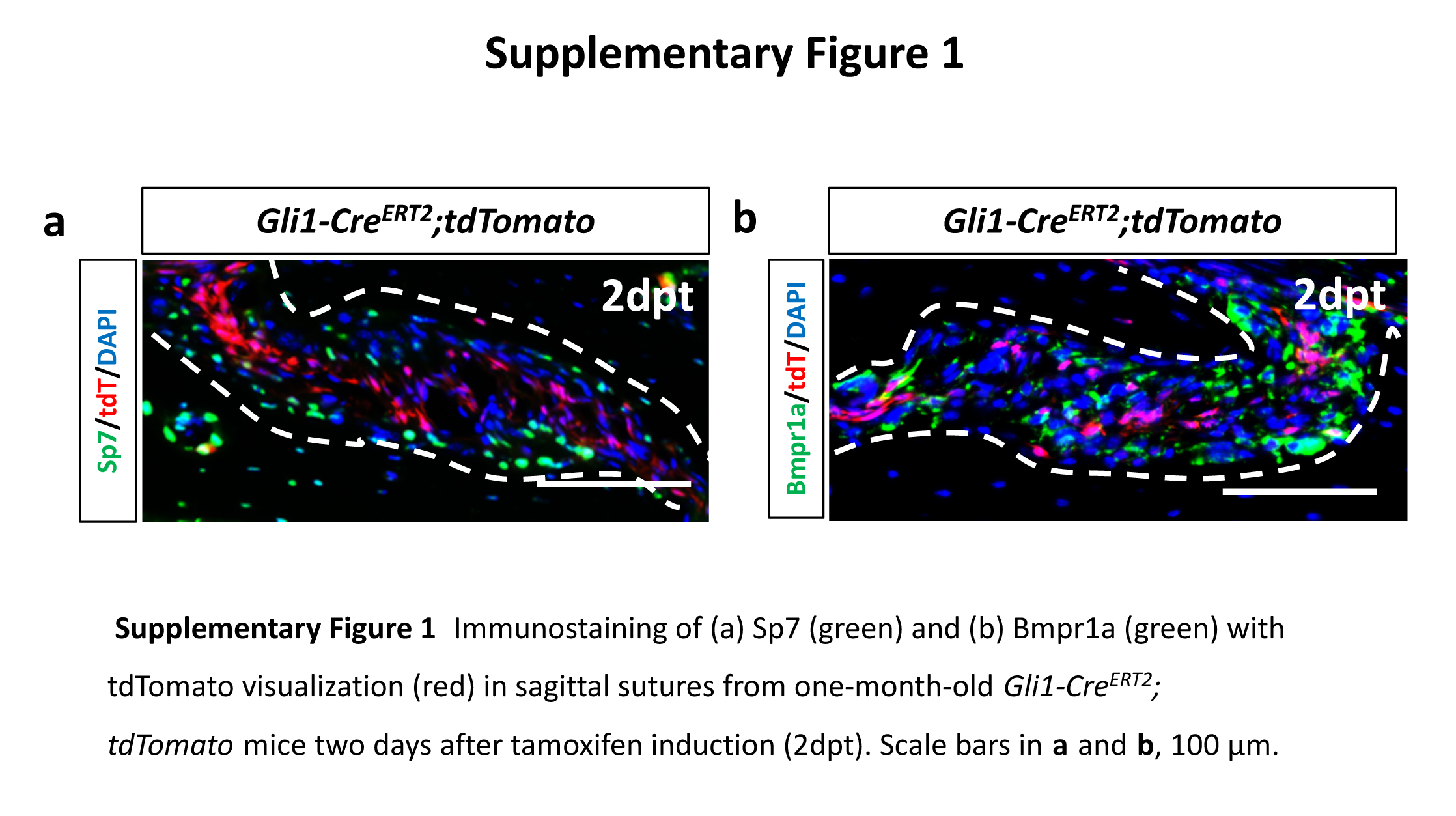

Supplement: Supplementary file 1 — Supplementary figure 1 [file 41413_2018_31_MOESM1_ESM.jpg]

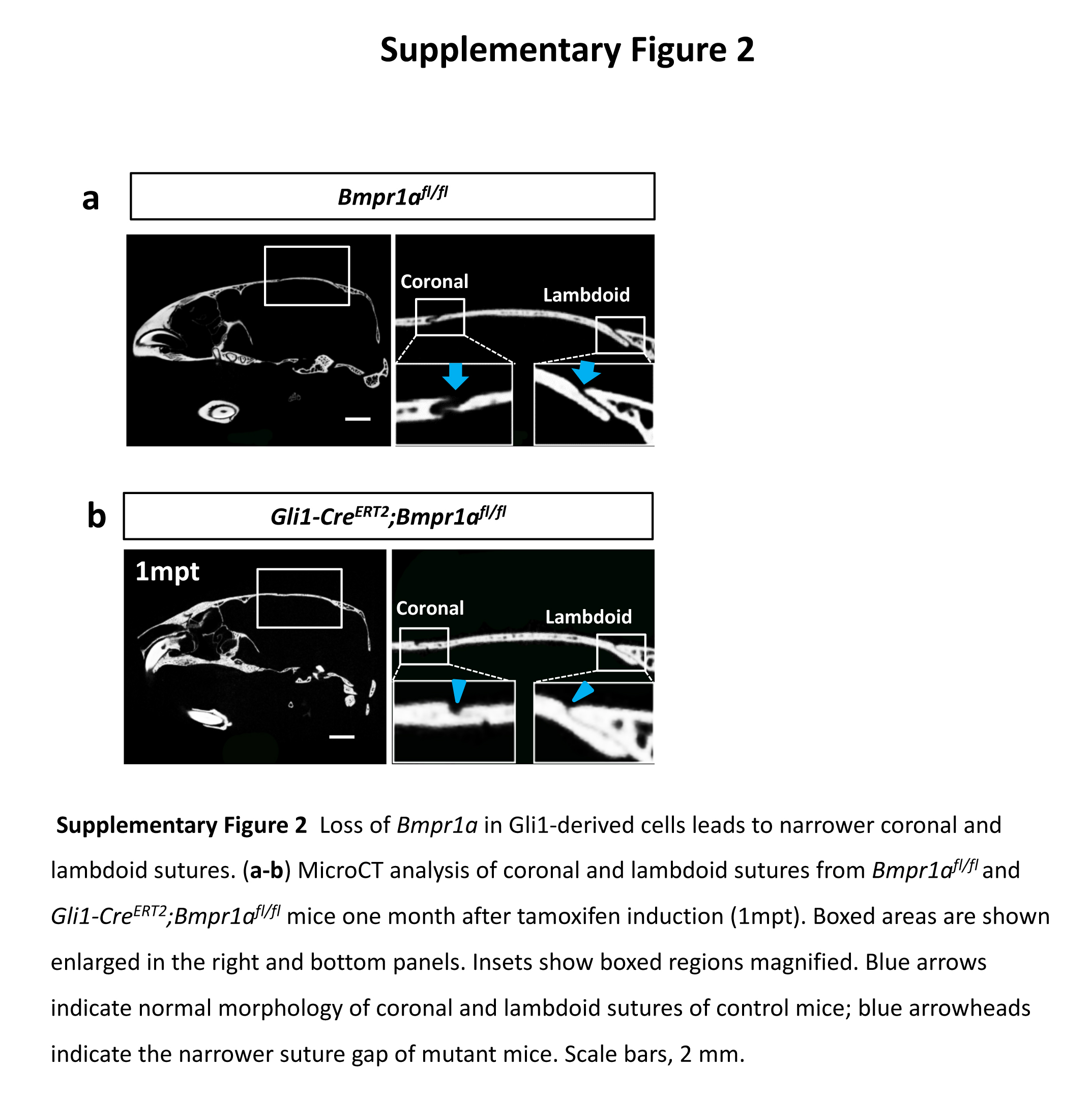

Supplement: Supplementary file 2 — Supplementary figure 2 [file 41413_2018_31_MOESM2_ESM.jpg]

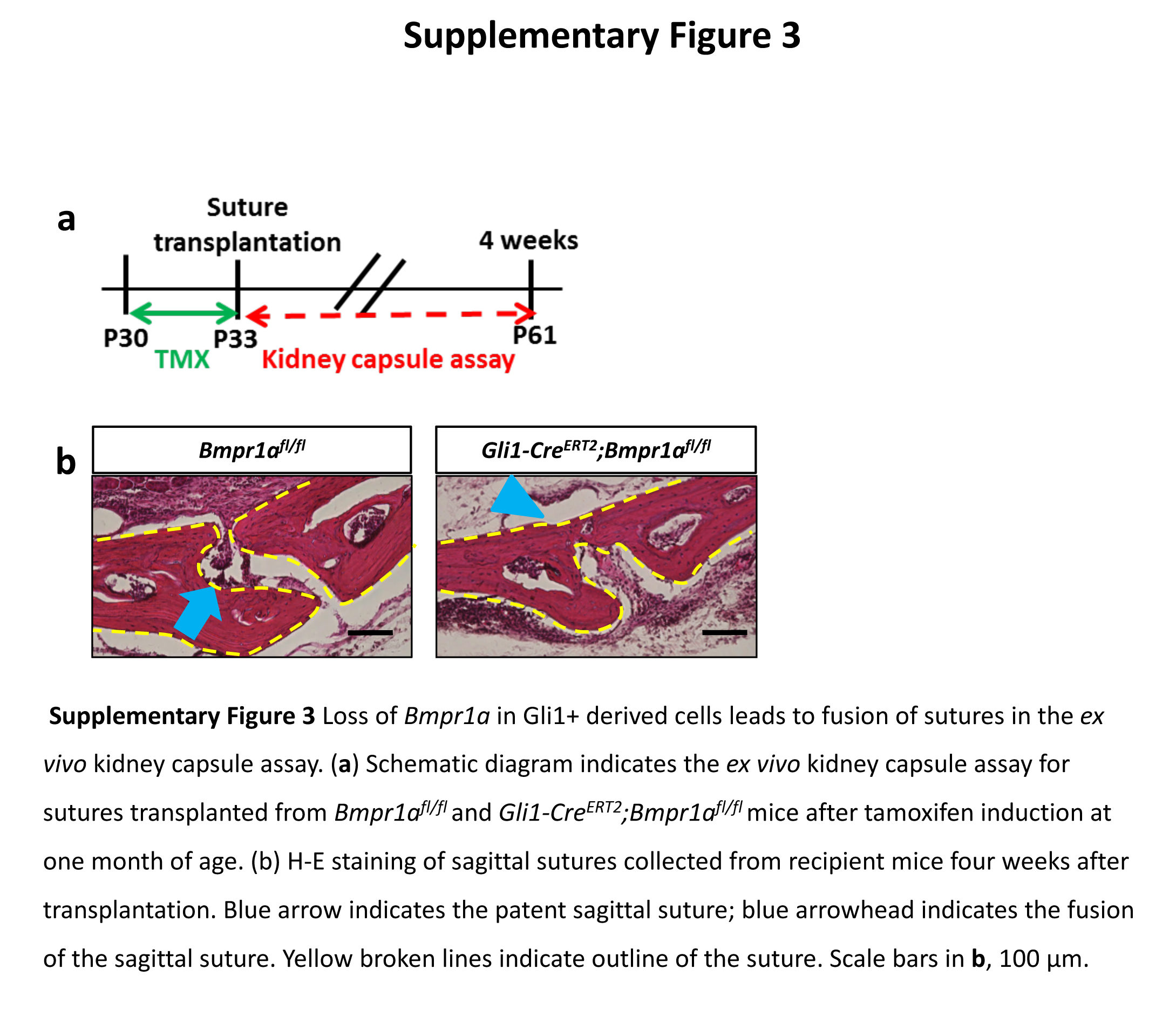

Supplement: Supplementary file 3 — Supplementary figure 3 [file 41413_2018_31_MOESM3_ESM.jpg]

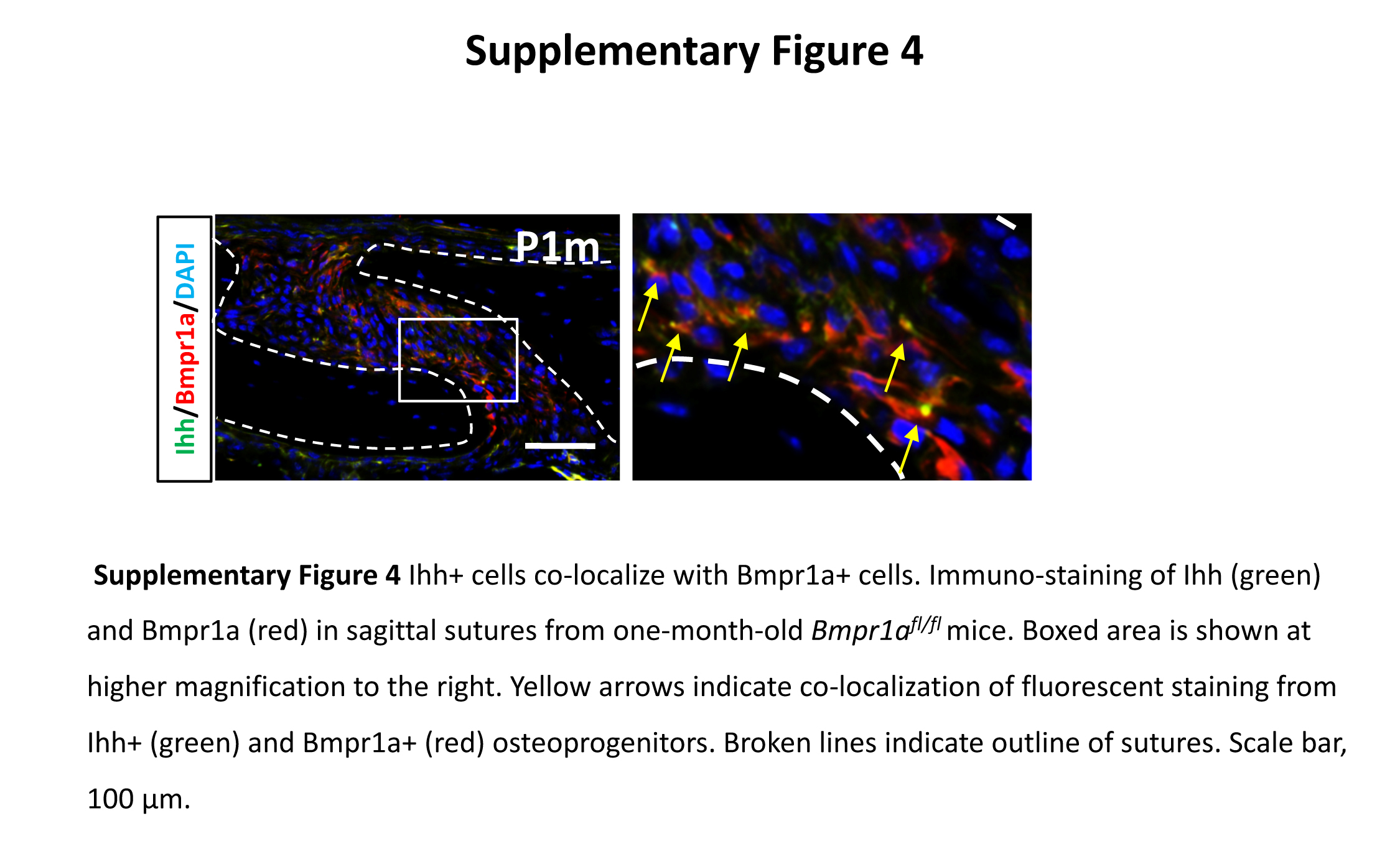

Supplement: Supplementary file 4 — Supplementary figure 4 [file 41413_2018_31_MOESM4_ESM.jpg]

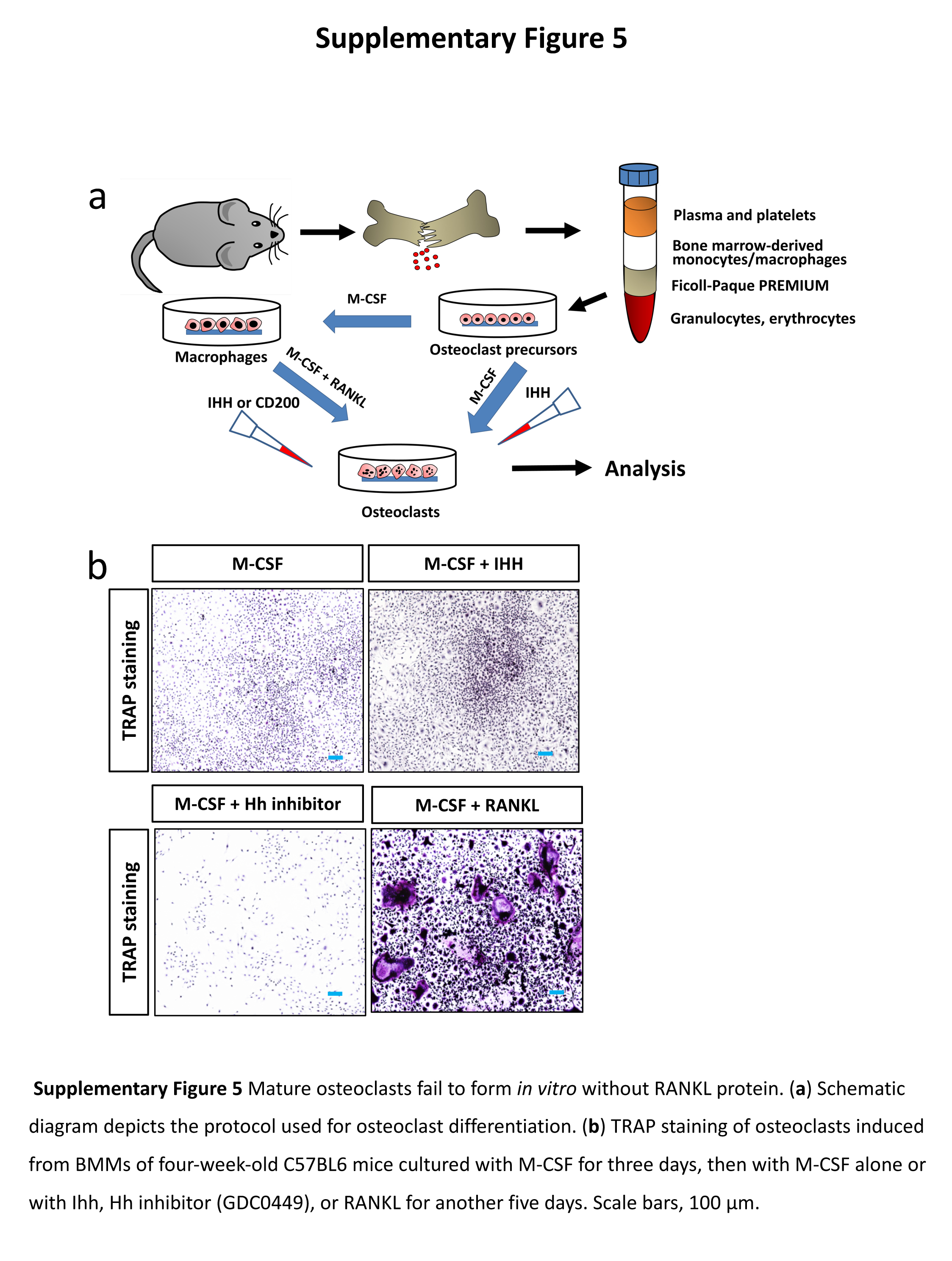

Supplement: Supplementary file 5 — Supplementary figure 5 [file 41413_2018_31_MOESM5_ESM.jpg]

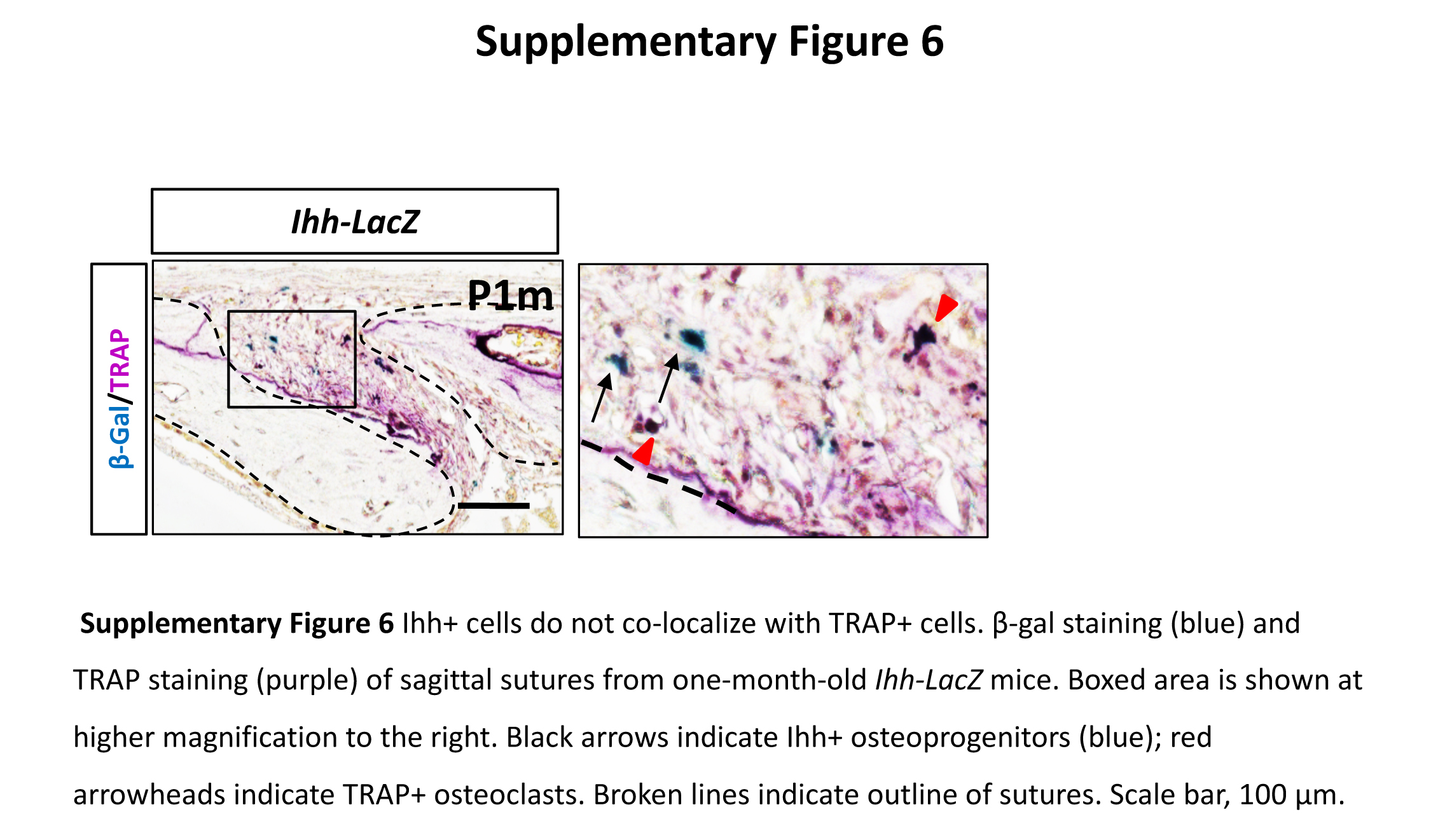

Supplement: Supplementary file 6 — Supplementary figure 6 [file 41413_2018_31_MOESM6_ESM.jpg]
